# What is the impact of one’s chronic illness on his or her spouse’s future chronic illness: a community-based prospective cohort study

**DOI:** 10.1186/s12916-023-03061-9

**Published:** 2023-10-16

**Authors:** Hoyoung An, Hee Won Yang, Dae Jong Oh, Eunji Lim, Jin Shin, Dong Gyu Moon, Seung Wan Suh, Seonjeong Byun, Tae Hui Kim, Kyung Phil Kwak, Bong Jo Kim, Shin Gyeom Kim, Jeong Lan Kim, Seok Woo Moon, Joon Hyuk Park, Seung-Ho Ryu, Dong Woo Lee, Seok Bum Lee, Jung Jae Lee, Jin Hyeong Jhoo, Jong Bin Bae, Ji Won Han, Ki Woong Kim

**Affiliations:** 1 Department of Neuropsychiatry, Keyo Hospital, Uiwang-Si, South Korea; 2 Department of Neuropsychiatry, Seoul National University Bundang Hospital, Seongnam, South Korea; 3 Workplace Mental Health Institute, Kangbuk Samsung Hospital, Sungkyunkwan University School of Medicine, Seoul, South Korea; 4 Department of Psychiatry, Gyeongsang National University Changwon Hospital, Changwon, South Korea; 5 Seoul Heal Mental Health Clinic, Seoul, South Korea; 6 Department of Psychiatry, Uijeongbu St. Mary’s Hospital, College of Medicine, The Catholic University of Korea, Uijeongbu, South Korea; 7 Department of Psychiatry, Yonsei University Wonju Severance Christian Hospital, Wonju, South Korea; 8 Department of Psychiatry, Dongguk University Gyeongju Hospital, Gyeongju, South Korea; 9 Department of Psychiatry, Gyeongsang National University School of Medicine, Jinju, South Korea; 10 Department of Neuropsychiatry, Soonchunhyang University Bucheon Hospital, Bucheon, South Korea; 11 Department of Psychiatry, School of Medicine, Chungnam National University, Daejeon, South Korea; 12 Department of Psychiatry, School of Medicine, Konkuk University, Konkuk University Chungju Hospital, Chungju, South Korea; 13 Department of Neuropsychiatry, Jeju National University Hospital, Jeju, South Korea; 14 Department of Psychiatry, School of Medicine, Konkuk University, Konkuk University Medical Center, Seoul, South Korea; 15 Department of Neuropsychiatry, Inje University Sanggye Paik Hospital, Seoul, South Korea; 16 Department of Psychiatry, Dankook University Hospital, Cheonan, South Korea; 17 Department of Psychiatry, Kangwon National University, School of Medicine, Chuncheon, South Korea; 18 Department of Brain and Cognitive Science, Seoul National University College of Natural Sciences, Seoul, South Korea; 19 Department of Psychiatry, Seoul National University, College of Medicine, Seoul, South Korea

**Keywords:** Chronic disease, Disease management, Spouse, Aged, Geriatrics

## Abstract

**Background:**

Integrating a joint approach to chronic disease management within the context of a couple has immense potential as a valuable strategy for both prevention and treatment. Although spousal concordance has been reported in specific chronic illnesses, the impact they cumulatively exert on a spouse in a longitudinal setting has not been investigated. We aimed to determine whether one’s cumulative illness burden has a longitudinal impact on that of their spouse.

**Methods:**

Data was acquired from a community-based prospective cohort that included Koreans aged 60 years and over, randomly sampled from 13 districts nationwide. Data from the baseline assessment (conducted from November 2010 to October 2012) up to the 8-year follow-up assessment was analyzed from October 2021 to November 2022. At the last assessment, partners of the index participants were invited, and we included 814 couples in the analysis after excluding 51 with incomplete variables. Chronic illness burden of the participants was measured by the Cumulative Illness Rating Scale (CIRS). Multivariable linear regression and causal mediation analysis were used to examine the longitudinal effects of index chronic illness burden at baseline and its change during follow-up on future index and spouse CIRS scores.

**Results:**

Index participants were divided based on baseline CIRS scores (CIRS < 6 points, *n* = 555, mean [SD] age 66.3 [4.79] years, 43% women; CIRS ≥ 6 points, *n* = 259, mean [SD] age 67.7 [4.76] years, 36% women). The baseline index CIRS scores and change in index CIRS scores during follow-up were associated with the spouse CIRS scores (*β* = 0.154 [SE: 0.039], *p* < 0.001 for baseline index CIRS; *β* = 0.126 [SE: 0.041], *p* = 0.002 for change in index CIRS) at the 8-year follow-up assessment. Subgroup analysis found similar results only in the high CIRS group. The baseline index CIRS scores and change in index CIRS scores during follow-up had both direct and indirect effects on the spouse CIRS scores at the 8-year follow-up assessment.

**Conclusions:**

The severity and course of one’s chronic illnesses had a significant effect on their spouse’s future chronic illness particularly when it was severe. Management strategies for chronic diseases that are centered on couples may be more effective.

**Supplementary Information:**

The online version contains supplementary material available at 10.1186/s12916-023-03061-9.

## Background

Chronic diseases are a heavy burden on public health. In 2019, nine of the top ten causes of global disease burden measured in disability-adjusted life-years (DALY) in the 50 to 74 years of age group, and eight of the top ten in the 75 years or older group were chronic diseases. And this is not a problem limited to developed countries anymore; the loss of life-years due to these causes has increased globally as improved health systems have led to longer life expectancies [1]. They are also a major cause of mortality worldwide. They accounted for nearly three-quarters of total global deaths in 2017, and their numbers are rising, with 7.6 million additional deaths attributed to them compared to a decade earlier [2]. These developments suggest that the management of chronic diseases is one of the main challenges facing clinicians worldwide.

The multimorbidity observed in patients with chronic diseases is cause for further concern. More than 60% of individuals between the ages of 18 and 44 who are seen in primary care have more than one condition, and the prevalence increases to more than 90% in older populations [3]. This can induce more complexity into treatment regimens, as they may need specifically targeted interventions, making effective management much more difficult.

To tackle this challenge, innumerable studies have been carried out to find the interventions, behaviors, and dietary patterns needed to improve the life expectancy for individuals living with a chronic disease, the results of which governments have promoted through health policies [4]. As a result, while there is no shortage of healthy advice one might heed, and an abundance of literature supporting them, actual implementation can be daunting for individuals [5–7]. Therefore, a way to improve the effectiveness and adherence to treatment without introducing more complexity is needed.

Approaching chronic diseases from a dyadic perspective, and viewing a couple as a team, whose main aim is to improve the health of both individuals may be an efficient way to improve the efficacy of and adherence to healthy behavior and treatment. Previous studies show that this may be true. Spouses not only show concordance in health-related behaviors [8, 9], but can influence adherence to treatment, dietary behavior, and physical activity [10–12]. This may be the reason why several risk factors for chronic diseases [9], and the diseases themselves also exhibit spousal concordance [13–15]. However, whether this is true for most chronic diseases, and continues to exert an effect over longer durations has not been investigated. The dearth of supporting evidence could potentially account for the limited availability of treatment approaches that specifically target couples as a unit rather than focusing solely on individuals. In considering both partners as current and potential patients, the scarcity of such strategies becomes apparent. One example of a dyadic intervention approach aligned with this perspective is a program designed to prevent type 2 diabetes. This program, developed by Whitaker et al., involves enrolling both individuals within a couple and fostering the adoption of healthy behaviors jointly [16].

In this study, we had two hypotheses. First, we hypothesized that an individual’s cumulative disease burden would influence his or her spouse’s cumulative disease burden. Second, we hypothesized that the change in an individual’s cumulative illness burden over time would independently influence the future cumulative illness burden of his or her spouse as well as himself or herself, regardless of the specific type of chronic illness.

## Methods

### Study design and participants

The index participants were the community-dwelling older adults who participated in the Korean Longitudinal Study on Cognitive Aging and Dementia (KLOSCAD) [17]. The KLOSCAD is a community-based nationwide prospective cohort study in which Koreans aged 60 years and over were randomly sampled from 13 districts across South Korea using residential rosters. The baseline assessment of the KLOSCAD was conducted from November 2010 to October 2012, and four biennial follow-up assessments have been completed. At the 8-year follow-up assessment, we invited the spouses of the index participants to the KLOSCAD, and 865 spouses responded. Among the 865 couples who responded to the 8-year follow-up assessment, we included 814 couples in the final analysis after excluding 51 couples whom either index or spouse participant failed to complete the assessments of the key variables included in the current analysis. All couples were formally married and living together.

All procedures involved in this study were approved by the Institutional Review Board (IRB) of the Seoul National University Bundang Hospital (IRB No. B-0912–089-010). In all cases, the study protocol and a detailed explanation were provided, and a written statement of informed consent was obtained, from either the participants or their legal guardians. This report followed the Strengthening the Reporting of Observational Studies in Epidemiology (STROBE) reporting guideline for cohort studies.

### Assessment of cumulative illness burden

We evaluated the cumulative illness burden of chronic illnesses using the Cumulative Illness Rating Scale (CIRS) [18]. The CIRS was developed as a simple, yet reliable measure of chronic physical and mental illnesses. It includes 14 organ systems, and the severity and/or impairment of each system is rated on a five-point severity scale. Smoking and obesity are also included and rated. The ratings from all systems can be summed into a global CIRS score, which we used in the current analysis. We used the modified version of the CIRS as reported by Miller and colleagues in 1992 [18], rather than the original version introduced by Linn et al. in 1968 [19]. The decision to use the modified version was based on the fact that the work of Miller et al. did not have the reporting bias identified in the study of Linn et al. [20]. In addition, the modified version of the CIRS has been validated in various populations [21, 22].

### Assessment of covariates

Educational attainment was measured as years of public education received and included graduate school.

To estimate the average lifetime alcohol use in standard units per week (SU/wk), we collected information from participants regarding their average weekly alcohol consumption in standard units and the total duration of alcohol consumption in years. Heavy alcohol use was defined as consuming more than 21 SU/wk on average throughout their lifetime.

Sleep quality was assessed using the Pittsburgh Sleep Quality Index (PSQI) [23]. The PSQI is a widely used self-report questionnaire that measures sleep quality and disturbance. It consists of 19 items that can be combined to produce a global score. Higher global scores indicate poorer self-reported sleep quality. For our analysis, we used the global PSQI scores.

To evaluate the level of physical activity among participants, we employed the Metabolic Equivalent Task (MET) [24]. We assessed the duration (minutes/day) and frequency (days/week) of light, moderate and vigorous activities. Light activities such as slow walking or dancing correspond to 3 METs, moderate activities such as fast walking or slow swimming correspond to 4.5 METs, and vigorous activities such as jogging, or tennis correspond to 8 METs. We provided participants with detailed examples of light, moderate, and vigorous activities to improve the accuracy of their reports. We estimated the total amount of physical activity per week by summing the total amount of energy expended through all reported activities in a week in MET-minutes.

We assessed the severity of depressive symptoms using the Geriatric Depression Scale (GDS) [25] and global cognitive function using the Korean version of the Mini-Mental Status Examination (MMSE) [26].

### Statistical analysis

First, we compared the demographic and clinical variables of the participants. For this, we classified the index participants into two groups using their CIRS score at the baseline assessment; the low cumulative illness burden (CIB) group whose baseline CIRS score was below six points, and the high CIB group whose baseline CIRS score was six points or higher.

To examine whether there were significant differences in demographic and clinical characteristics between the low and high CIB groups that might influence future CIB in themselves and their spouses, we conducted a series of comparisons. We used Student’s *t*-tests to examine differences between groups for continuous variables and chi-squared tests for categorical variables. These comparisons were made separately for each assessment and for index and spouse cases. We used paired *t*-tests for continuous variables and McNemar’s chi-square tests for categorical variables to examine differences between the baseline and the 8-year follow-up assessments within each group.

Second, we conducted cross-sectional analyses to determine whether the CIB of the index and spouse participants were correlated with each other. We used Pearson’s correlation and partial correlation analysis. The latter analysis controlled for age, sex, years of education, exercise, heavy alcohol use, PSQI score, and GDS score of the spouse participants.

Third, we conducted multiple linear regression analyses to investigate the effects of index CIB at the baseline assessment and its change during the follow-up period on both index and spouse CIB at the 8-year follow-up assessment. We used multiple linear regression analyses that computed the baseline index CIRS score and the change in index CIRS score between the baseline and the 6-year follow-up assessment (ΔCIRS) as independent variables, either the index or spouse CIRS scores at the 8-year follow-up assessment as dependent variables, and age, sex, years of education, exercise, heavy alcohol use, PSQI score, and GDS score at the corresponding assessment as covariates. We did not adjust for smoking and body mass index in these analyses because they were included in the CIRS.

Finally, we examined how the change in index CIB mediated the effects of baseline index CIB on index and spouse CIB at the 8-year follow-up assessment using causal mediation analysis. Causal mediation analysis is a statistical technique that estimates the role of a mediator in a potentially causal relationship between two variables. This was repeated separately for the low and high CIB groups. Bootstrapping with 2000 iterations was used for all causal mediation analyses.

We performed all statistical analyses using R version 4.1.2 (R Core Team) and the dplyr and mediation packages.

## Results

### Demographic and clinical characteristics

As summarized in Table 1, the index participants in the low CIB group were slightly younger (66.3 years vs 67.7 years), exercised more (2244 MET × min/week vs 1768 MET × min/week), and drank more (5% vs 2%) but were slightly less educated (10.1 years vs 10.8 years), less depressive (8.0 points vs 10.0 points on the GDS), and had better self-reported sleep quality (5.5 points vs 6.5 points on the PSQI) than those in the high CIB group at the baseline assessment. During the follow-up period (8.11 ± 0.3 years), in the index participants, the amount of exercise (2093 to 1437 MET × min/week) and depression scores (8.6 points to 8.1 points on the GDS) decreased while the CIRS score increased (4.4 points to 6.7 points). Index participants in the low CIB group also showed increased PSQI scores (5.5 points to 5.9 points) at the 8-year follow-up assessment.

**Table 1:** Characteristics of the participants

	Baseline assessment			8-year follow-up assessment			Statistics			
	All (*n* = 814)	LCIB^a^ (*n* = 555)	HCIB^b^ (*n* = 259)	All (*n* = 814)	LCIB^c^ (*n* = 555)	HCIB^d^ (*n* = 259)	a vs b^*^	c vs d^*^	a vs c^†^	b vs d^†^
Age, years										
Index	66.7 (4.82)	66.3 (4.79)	67.7 (4.76)	74.8 (4.82)	74.4 (4.79)	75.7 (4.76)	0.001	0.001	< 0.001	< 0.001
Spouse	-	-	-	73.6 (6.01)	73.4 (6.03)	73.9 (5.98)	-	0.24	-	-
*p*^‡^	-	-	-	< 0.001	0.003	< 0.001	-	-	-	-
Women										
Index	331 (40.7)	238 (42.9)	93 (35.9)	331 (40.7)	238 (42.9)	93 (35.9)	0.06	0.06	1.00	1.00
Spouse	-	-	-	483 (59.3)	317 (57.1)	166 (64.1)	-	0.06	-	-
*p*^‡^	-	-	-	< 0.001	< 0.001	< 0.001	-	-	-	-
Education, years										
Index	10.3 (4.90)	10.1 (4.90)	10.8 (4.87)	10.3 (4.90)	10.1 (4.90)	10.8 (4.87)	0.04	0.04	1.00	1.00
Spouse	-	-	-	10.0 (4.98)	9.8 (5.03)	10.5 (4.82)	-	0.04	-	-
*p*^‡^	-	-	-	0.20	0.28	0.49	-	-	-	-
Exercise, MET*min/week										
Index	2093 (3295)	2244 (3556)	1768 (2629)	1437 (2092)	1550 (2343)	1195 (1382)	0.03	0.007	< 0.001	< 0.001
Spouse	-	-	-	1294 (1802)	1395 (1966)	1075 (1365)	-	0.007	-	-
*p*^‡^	-	-	-	0.14	0.24	0.32	-	-	-	-
CIRS, points										
Index	4.4 (2.85)	2.9 (1.55)	7.7 (2.10)	6.7 (3.35)	5.8 (2.95)	8.9 (3.16)	< 0.001	< 0.001	< 0.001	< 0.001
Spouse	-	-	-	5.7 (3.15)	5.5 (3.06)	6.1 (3.31)	-	0.02	-	-
*p*^‡^	-	-	-	< 0.001	0.14	< 0.001	-	-	-	-
High CIRS^§^										
Index	259 (31.8)	0 (0.0)	259 (100.0)	491 (60.3)	268 (48.3)	223 (86.1)	< 0.001	< 0.001	< 0.001	< 0.001
Spouse	-	-	-	387 (47.5)	248 (44.7)	139 (53.7)	-	0.02	-	-
*p*^‡^	-	-	-	< 0.001	0.23	< 0.001	-	-	-	-
Heavy alcohol use^¶^										
Index	34 (4.2)	28 (5.0)	6 (2.3)	34 (4.2)	28 (5.0)	6 (2.3)	0.070	0.070	1.00	1.00
Spouse	-	-	-	30 (3.7)	26 (4.7)	4 (1.5)	-	0.03	-	-
*p*^‡^	-	-	-	0.61	0.78	0.52	-	-	-	-
PSQI, points										
Index	5.8 (3.14)	5.5 (2.94)	6.5 (4.40)	6.0 (3.32)	5.9 (3.34)	6.1 (3.30)	< 0.001	0.29	0.014	0.15
Spouse	-	-	-	6.0 (3.52)	5.8 (3.47)	6.5 (3.60)	-	0.02	-	-
*p*^‡^	-	-	-	0.70	0.79	0.29	-	-	-	-
GDS, points										
Index	8.6 (5.98)	8.0 (5.83)	10.0 (6.10)	8.1 (6.21)	7.5 (5.84)	9.4 (6.77)	< 0.001	< 0.001	0.059	0.24
Spouse	-	-	-	8.4 (6.26)	8.0 (6.0)	9.3 (6.73)	-	0.010	-	-
*p*^‡^	-	-	-	0.37	0.17	0.78	-	-	-	-
MMSE, points										
Index	27.0 (2.64)	27.0 (2.50)	27.0 (2.92)	27.0 (2.95)	27.0 (2.85)	27.0 (3.15)	0.93	0.87	0.91	0.85
Spouse	-	-	-	26.4 (3.30)	26.3 (3.22)	26.6 (3.46)	-	0.34	-	-
*p*^‡^		-	-	< 0.001	< 0.001	0.12	-	-	-	-

Continuous variables are presented as mean (standard deviation) and categorical variables as number (percentage)

*LCIB* low cumulative illness burden (CIRS < 6), *HCIB* high cumulative illness burden (CIRS ≥ 6), *MET* Metabolic Equivalent of Task, *CIRS* Cumulative Illness Rating Scale, *PSQI* Pittsburg Sleep Quality Index, *GDS* Geriatric Depression Scale, *MMSE* Mini-Mental State Examination

^*^Student’s t-test for continuous variables and chi-square tests for categorical variables between the LCIB group and the HCIB group

^†^Paired t-test for continuous variables and McNemar’s chi-square tests for categorical variables between the baseline and 8-year follow-up assessments within the index participants

^‡^Student’s t-test for continuous variables and chi-square tests for categorical variables between the index participants and the spouse participants at the 8-year follow-up assessment

^§^CIRS score of six points or higher at the 8-year follow-up assessment

^¶^Average lifetime alcohol use of greater than 21 standard units per week

At the 8-year follow-up assessment, the index participants were slightly older (74.8 years vs 73.6 years), less likely to be women (41% vs 59%), and had higher CIRS scores (6.7 points vs 5.7 points) than the spouse participants. In both index and spouse participants, the low CIB group was less educated (index participants, 10.1 years vs 10.8 years; spouse participants, 9.8 years vs 10.5 years), less depressive (index participants, 7.5 points vs 9.4 points; spouse participants, 8.0 points vs 9.3 points), and exercised more (index participants, 1,550 MET × min/week vs 1195 MET × min/week; spouse participants, 1395 MET × min/week vs 1,075 MET × min/week) than the high CIB group. In the index participants, the low CIB group was younger (74.4 years vs 75.7 years) and more likely to be women (43% vs 36%) than the high CIB group. In the spouse participants, the spouses of the index participants with low CIB had lower CIRS scores (5.5 points vs 6.1 points) and PSQI scores (5.8 points vs 6.5 points) than those of the index participants with high CIB, indicating that the spouses of the index participants with low CIB also had lower CIRS scores and better self-reported sleep quality than those of the index participants with high CIB (Table 1).

Demographic and clinical characteristics of the index participants who participated in the couple cohort and those who did not, and couples who were included in the analysis and those who were not are compared in the Additional file 1: Tables S1 and S2. Index participants who participated in the couple cohort were younger, more educated, and had better MMSE scores than those who did not.

### Associations of index CIB with future index CIB and spouse CIB

At the 8-year follow-up assessment, the index CIRS score was correlated with the spouse CIRS score (unadjusted correlation coefficient = 0.157, *p* < 0.001; adjusted correlation coefficient = 0.155, *p* < 0.001 for adjusted model). As shown in Table 2, the baseline index CIRS score and the change in index CIRS score during the follow-up period were associated with both the index CIRS score (*β* = 0.841 [SE 0.023], *p* < 0.001 for baseline index CIRS score; *β* = 0.783 [SE 0.025], *p* < 0.001 for the changes in index CIRS score) and the spouse CIRS score (*β* = 0.154 [SE 0.039], *p* < 0.001 for baseline index CIRS score; *β* = 0.126 [SE 0·0.041], *p* = 0.002 for the changes in index CIRS score) at the 8-year follow-up assessment. These findings indicate that for each one-point increase in the baseline index CIRS score, there was a corresponding increase of 0.841 points in the index participants’ CIRS score and a 0.154-point increase in their spouses’ CIRS score at the 8-year follow-up assessment. Similarly, each one-point increase in the change in index CIRS score was associated with a 0.783-point increase in the index participants’ CIRS score and a 0.126-point increase in their spouses’ CIRS score at the 8-year follow-up assessment. When we conducted separate analyses for the low and high CIB groups, we obtained similar findings in the high CIB group. In the high CIB group, both the baseline index CIRS score and the change in index CIRS score during the follow-up period were significantly associated with the index CIRS score (*β* = 0.759 [SE 0.056], *p* < 0.001 for baseline index CIRS score; *β* = 0.768 [SE 0.044], *p* < 0.001 for the change in index CIRS score) and the spouse CIRS score (*β* = 0.293 [SE 0.093], *p* = 0.002 for baseline index CIRS score; *β* = 0.218 [SE 0.076], *p* = 0.005 for the change in index CIRS score) at the 8-year follow-up assessment (Table 2).

**Table 2:** Longitudinal effects of the index participant cumulative illness burden at baseline and its change during follow-up on that of the index participants and their spouses at the 8-year follow-up assessment

	CIRS at the 8-year follow-up assessment			
	Index^a^		Spouse^b^	
	Estimate (SE)	*p*	*Estimate (SE)*	*p*
All index participants				
Baseline index CIRS	0.841 (0.023)	< 0.001	0.154 (0.039)	< 0.001
Change in index CIRS	0.783 (0.025)	< 0.001	0.126 (0.041)	0.002
Index participants with low CIB^c^				
Baseline index CIRS	0.916 (0.048)	< 0.001	0.102 (0.080)	0.200
Change in index CIRS	0.789 (0.030)	< 0.001	0.091 (0.049)	0.062
Index participants with high CIB^d^				
Baseline index CIRS	0.759 (0.056)	< 0.001	0.293 (0.093)	0.002
Change in index CIRS	0.768 (0.044)	< 0.001	0.218 (0.076)	0.005

*CIB* cumulative illness burden, *CIRS* Cumulative Illness Rating Scale, *SE* standard error

^a^Multiple linear regression analyses adjusting for age, sex, years of education, exercise and Geriatric Depression Scale score of the index participants at the 8-year follow-up assessment. Estimates are unstandardized

^b^Multiple linear regression analyses adjusting for age, sex, years of education, exercise, heavy alcohol use, Pittsburg Sleep Quality Index score, and Geriatric Depression Scale score of the spouse participants at the 8-year follow-up assessment. Estimates are unstandardized

^c^Baseline index CIRS score of below six points

^d^Baseline index CIRS score of six points or higher

### Effects of index CIB on future index CIB and spouse CIB

**Fig. 1:**
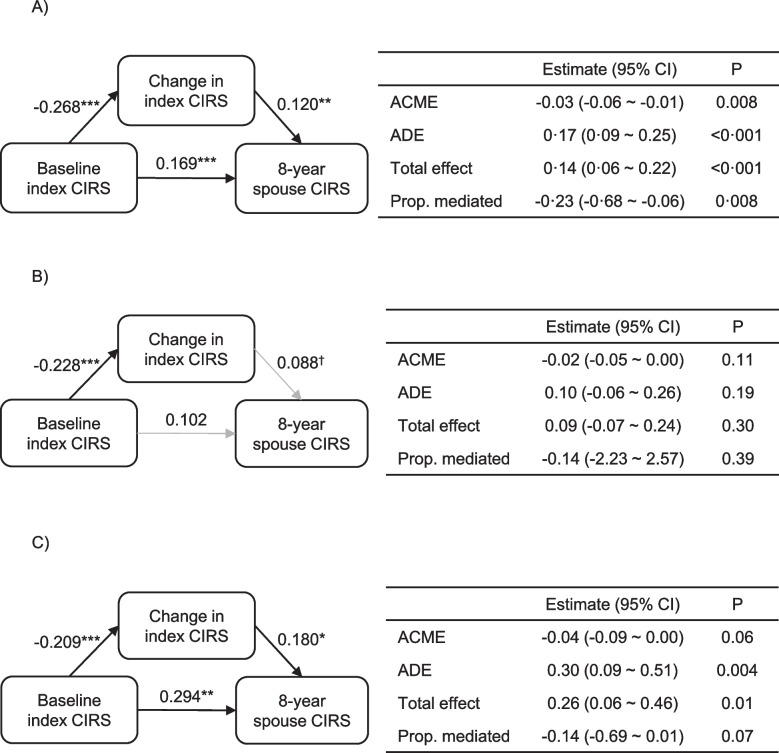
The effects of index cumulative illness burden on future spouse cumulative illness burden. Causal mediation analysis models examining the effects of index CIB (cumulative illness burden) at baseline and its change on future spouse CIB in **A** all subjects, **B** the low CIB group, and **C** the high CIB group. “Baseline index CIRS” refers to the CIRS scores of the index participants at the baseline visit. “Change in index CIRS” refers to the change in CIRS scores (ΔCIRS) of the index participants between the baseline visit and the 6-year follow-up visit. The spouse CIRS scores were assessed once, at the 8-year follow-up visit, and is presented as “8-year spouse CIRS.” Numbers next to arrows correspond to unstandardized regression coefficients. Black arrows indicate statistically significant effects. ACME, average causal mediation effect; ADE, average direct effect. ^†^*p* < .01, *p < .05, **p < .01, ***p < .001

In the mediation analyses, the baseline index CIRS score and the change in index CIRS score during the follow-up period showed both direct and indirect effects on the spouse CIRS score at the 8-year follow-up assessment, and subgroup analysis with index participants with high baseline CIB yielded similar results (Fig. 1). These results suggest that the change in the index CIRS score not only directly influences the spouse CIRS score, as indicated by the linear regression analyses, but also acts as a mediator of the effect of the baseline index CIRS score. In the latter analysis, the baseline index CIRS score had an indirect effect on spouse CIRS score that was mediated by the change in index CIRS score (unstandardized indirect effect: − 0.04, 95% CI: − 0.09 ~ 0.00, *p* = 0.062) that neared statistical significance. The baseline index CIRS score also had a direct effect on the spouse CIRS score (unstandardized direct effect: 0.30, 95% CI: 0.09 ~ 0.51, *p* = 0.004). The indirect effect constituted 14% (*p* = 0.074) of the total effect of baseline index CIRS score on spouse CIRS score at the 8-year follow-up assessment. The results of the mediation analysis examining the effects of baseline index CIRS score and the change in index CIRS score during the follow-up period on the index CIRS score at the 8-year follow-up assessment is presented as Additional file 1: Fig. S1.

## Discussion

This study demonstrated that CIB was highly concordant within older couples. Although the spouses’ CIB was assessed only once, it was found that both the initial level and trajectory of an individual’s CIB had a significant influence on their own CIB and potentially on their spouse’s CIB as well. These results suggest that a couple-based dyadic approach may be warranted in managing chronic illnesses of older adults.

Some chronic illnesses such as diabetes mellitus, coronary heart disease, stroke, depression, and dementia are shared between couples [13–15]. For example, one’s diabetes mellitus can increase the risk of diabetes by up to 40% in his or her spouse [13]. An individual’s dementia, stroke, or depression can also increase the risks of them in his or her spouse [15, 27, 28]. However, such spousal concordance is not common in most types of cancer [29], suggesting that genetic contributions may be stronger in cancer compared to other chronic illnesses.

The spousal concordance of chronic illnesses may be due to shared environmental and behavioral characteristics. In most cases, couples have a common environmental and socioeconomical status, show similar educational attainment [30], and can share similar eating habits and physical activity levels [9]. An individual’s behavior such as smoking and alcohol consumption can also influence the behaviors of his or her spouse [8]. These factors can then cause health-related biological changes such as high body mass index (BMI), high blood pressure, or high blood HDL cholesterol [9]. In the current study, the spouse CIB at the 8-year follow-up assessment was not only associated with the index CIB at the same assessment but also with the baseline index CIB and the change in index CIB. The direct effect of the baseline index CIB and the change in index CIB on spouse CIB was also significant in the mediation analyses. These results provide further evidence supporting the attributions that the long-term shared behavior and environment within couples may have on the spousal concordance of CIB. Based on these findings, the health of an individual within a couple may be considered a function of both individual factors (genetic factors, behavioral factors, and environmental factors before marriage) and shared factors (shared behavioral factors and shared environmental factors).

In the current study, the effects of the baseline index CIB and the change in index CIB on follow-up index CIB were significant in both low and high CIB groups. However, their effects on follow-up spouse CIB were significant in the high CIB group only. The baseline index CIB and the change in index CIB explained 26% of the spouse CIB at the follow-up assessment in the high CIB group but only 9% in the low CIB group. This suggests that when one’s CIB is low, the effects of the baseline CIB and the changes in CIB of the index participants may not be strong enough to influence the CIB of their spouses which is determined by their spouses’ genetic factors, unshared behaviors, and unshared environmental factors before their marriages. In addition, caregiver burden may also mediate the spousal concordance in CIB when one’s CIB is high. Disease severity is a well-known determinant of caregiver burden in several chronic conditions, and the presence of comorbidities and a patient’s functional status are also important deciding factors of caregiver burden [31]. Caregiver burden has been associated with dietary choices [32] and insufficient rest and time to exercise [33]. Although the dose–response relationship of spousal concordance in chronic illnesses has not been investigated much, the results of two previous studies are in line with those of the current study. Wang et al. found that one’s functional limitation increased his or her spouse’s functional limitations dose-dependently [34]. Nielsen et al. reported that one’s obesity increased the risk of type 2 diabetes dose-dependently in his or her spouse [35]. These studies also suggested that the higher one’s CIB, the stronger its effect on the CIB of his or her spouses’.

In our mediation models, the effect of the changes in index CIB was, although weaker than that of the baseline index CIB, significant on the spouse CIB at the follow-up assessment. In the index participants with high baseline CIB, the higher the baseline index CIB, the higher the spouse CIB at the follow-up assessment. The baseline index CIB explained 29.4% of the spouse CIB at the follow-up assessment. However, the more the index CIB reduced during the follow-up period, the lower the spouse CIB at the follow-up assessment. In the current sample, the change in the index CIB during the follow-up period explained 4% of the spouse CIB at the follow-up assessment. In previous studies, spouse functioning was the most pervasive determinant of patient functioning in cancer management [36] and the trajectories of health-related behaviors are often similar between couples [37]. These results indicate that a dyadic approach may be effective when managing chronic diseases in older couples.

However, it is important to consider the negative effect of the baseline index CIB on the change in index CIB. This observation may be attributed to the relatively good health status of our study subjects and the associated floor effect. In our community-based sample, the average CIRS score was 4.4 points, and 68% of the index participants had scores below 6 points at the baseline visit, which is lower compared to other studies [21, 22]. Consequently, the majority of individuals with lower CIRS scores were limited to experiencing an increase.

The strengths of this study lie in its long-term prospective follow-up period of 8 years and the utilization of a large sample consisting of community-dwelling older couples. To our knowledge, this is the first longitudinal study examining the reciprocal effects of one person’s cumulative illness burden on the other in couples. Most previous studies were either cross-sectional in design [8, 9, 13, 27], or focused on specific chronic diseases rather than the cumulative burden of multiple diseases [35, 37]. However, this study also has several limitations. First, we could not consider the baseline CIB of spouses in the current study because the couple cohort was established at the 8-year follow-up assessment of the index cohort and the CIB of spouses was assessed only once. As the KLOSCAD is ongoing, we plan to perform additional analyses examining the longitudinal interactions of change in CIB between spouses in the future. Second, we determined the CIB based on the reports of the participants and thus were subject to recall biases. However, considering that the MMSE scores of the participants were within the normal range, recall biases might not have been significant. Third, in addition to supporting the use of a dyadic perspective in the management of chronic illness, the aim of this study is to emphasize that further investigations are necessary for the widespread adoption of this approach. It is crucial to explore the complex ethical considerations associated with dyadic interventions. These considerations should encompass aspects such as marital quality, inter-relationship dynamics, and privacy.

## Conclusions

In conclusion, CIB was highly concordant within older couples and one’s severity and course of CIB had a significant effect on his or her spouse’s future CIB particularly when one’s current CIB was severe. Therefore, evaluation and management strategies for chronic diseases that are centered on couples rather than individuals in older adults may be a simple and effective way to improve the effectiveness of existing treatments.

### Supplementary Information

**Additional file 1: Table S1.** Comparison of baseline demographic and clinical characteristics between the participants of the Korean Longitudinal Study on Cognitive Aging and Dementia who were invited to the current study as index cases and those who were not. **Table S2.** Comparison of demographic and clinical characteristics at the 8-year follow-up assessment between the couples included in the final analysis and those who were excluded. **Figure S1.** Causal mediation analysis model showing the effects of index CIB at baseline and its change on future index CIB.

## Data Availability

The datasets used and analyzed during the current study are available from the corresponding author, Prof. K. W. Kim, on reasonable request.

## Abbreviations

CIB: Cumulative illness burden
CIRS: Cumulative Illness Rating Scale
GDS: Geriatric Depression Scale
KLOSCAD: Korean Longitudinal Study on Cognitive Aging and Dementia
MET: Metabolic Equivalent Task
MMSE: Mini-Mental Status Examination
PSQI: Pittsburg Sleep Quality Index
SU: Standard units of alcohol
