# Supplementary material for: What is the impact of one’s chronic illness on his or her spouse’s future chronic illness: a community-based prospective cohort study
Source: BMC Med. 2023 Oct 16;21:367. doi: 10.1186/s12916-023-03061-9 (PMC10578032; doi:10.1186/s12916-023-03061-9)

Table S1. Comparison of baseline demographic and clinical characteristics between the participants of the Korean Longitudinal Study on Cognitive Aging and Dementia who were invited to the current study as index cases and those who were not

|  | Invited  (n = 865) | Not invited  (n = 5,953) | P^*^ |
| --- | --- | --- | --- |
| Age, years | 66.9 (4.89) | 71.0 (7.21) | <0.001 |
| Women | 351 (40.6) | 3,568 (59.9) | <0.001 |
| Education, years | 10.2 (4.97) | 7.5 (5.34) | <0.001 |
| Exercise, MET × min/week | 2152 (3834) | 1305 (2604) | <0.001 |
| CIRS, points | 4.4 (2.85) | 4.5 (2.85) | 0.22 |
| High CIRS^†^ | 266 (30.8) | 1,860 (31.2) | 0.46 |
| Heavy alcohol use^‡^ | 38 (4.4) | 400 (6.7) | 0.006 |
| PSQI, points | 5.8 (3.47) | 6.5 (3.75) | <0.001 |
| GDS, points | 8.6 (6.04) | 10.3 (6.67) | <0.001 |
| MMSE, points | 26.9 (2.52) | 24.9 (4.47) | <0.001 |

MET, Metabolic Equivalent of Task; CIRS, Cumulative Illness Rating Scale; PSQI, Pittsburg Sleep Quality Index; GDS, Geriatric Depression Scale; MMSE, Mini-Mental State Examination

Note. Continuous variables are presented as mean (standard deviation) and categorical variables as number (percentage).

^*^ Student’s t-test for continuous variables and chi-square tests for categorical variables between the invited and non-invited participants

^†^ CIRS score of six points or higher

^‡^ Average lifetime alcohol use of greater than 21 standard units per week

Table S2. Comparison of demographic and clinical characteristics at the 8-year follow-up assessment between the couples included in the final analysis and those who were excluded

|  | Included  (n = 814 couples) | Not included  (n = 51 couples) | P^*^ |
| --- | --- | --- | --- |
| Age, years |  |  |  |
| Index | 74.8 (4.82) | 76.4 (5.68) | 0.05 |
| Spouse | 73.6 (6.01) | 75.8 (7.31) | 0.03 |
| Women |  |  |  |
| Index | 331 (40.7) | 20 (39.2) | 0.84 |
| Spouse | 483 (59.3) | 31 (60.8) | 0.84 |
| Education, years |  |  |  |
| Index | 10.3 (4.90) | 8.75 (5.83) | 0.06 |
| Spouse | 10.0 (4.98) | 8.47 (5.12) | 0.04 |
| Exercise, MET × min/week |  |  |  |
| Index | 1437 (2092) | 1009 (1495) | 0.06 |
| Spouse | 1294 (1802) | 1109 (1561) | 0.42 |
| CIRS, points |  |  |  |
| Index | 6.7 (3.35) | 6.0 (3.18) | 0.10 |
| Spouse | 5.7 (3.15) | 6.6 (3.58) | 0.08 |
| High CIRS^†^ |  |  |  |
| Index | 259 (31.8) | 13 (25.5) | 0.51 |
| Spouse | 387 (47.5) | 33 (64.7) | 0.02 |
| Heavy alcohol use^‡^ |  |  |  |
| Index | 34 (4.2) | 4 (7.8) | 0.22 |
| Spouse | 30 (3.7) | 3 (5.9) | 0.43 |
| PSQI, points |  |  |  |
| Index | 6.0 (3.32) | 5.5 (3.97) | 0.43 |
| Spouse | 6.0 (3.52) | 6.0 (3.64) | 0.98 |
| GDS, points |  |  |  |
| Index | 8.1 (6.21) | 8.39 (6.70) | 0.77 |
| Spouse | 8.4 (6.26) | 9.2 (6.64) | 0.42 |
| MMSE, points |  |  |  |
| Index | 27.0 (2.95) | 25.6 (3.73) | 0.009 |
| Spouse | 26.4 (3.30) | 25.0 (3.79) | 0.01 |

MET, Metabolic Equivalent of Task; CIRS, Cumulative Illness Rating Scale; PSQI, Pittsburg Sleep Quality Index; GDS, Geriatric Depression Scale; MMSE, Mini-Mental State Examination

Note. Continuous variables are presented as mean (standard deviation) and categorical variables as number (percentage).

^*^ Student’s t-test for continuous variables and chi-square tests for categorical variables between the participants included in the final analysis and those who were not included

^†^ CIRS score of six points or higher

^‡^ Average lifetime alcohol use of greater than 21 standard units per week


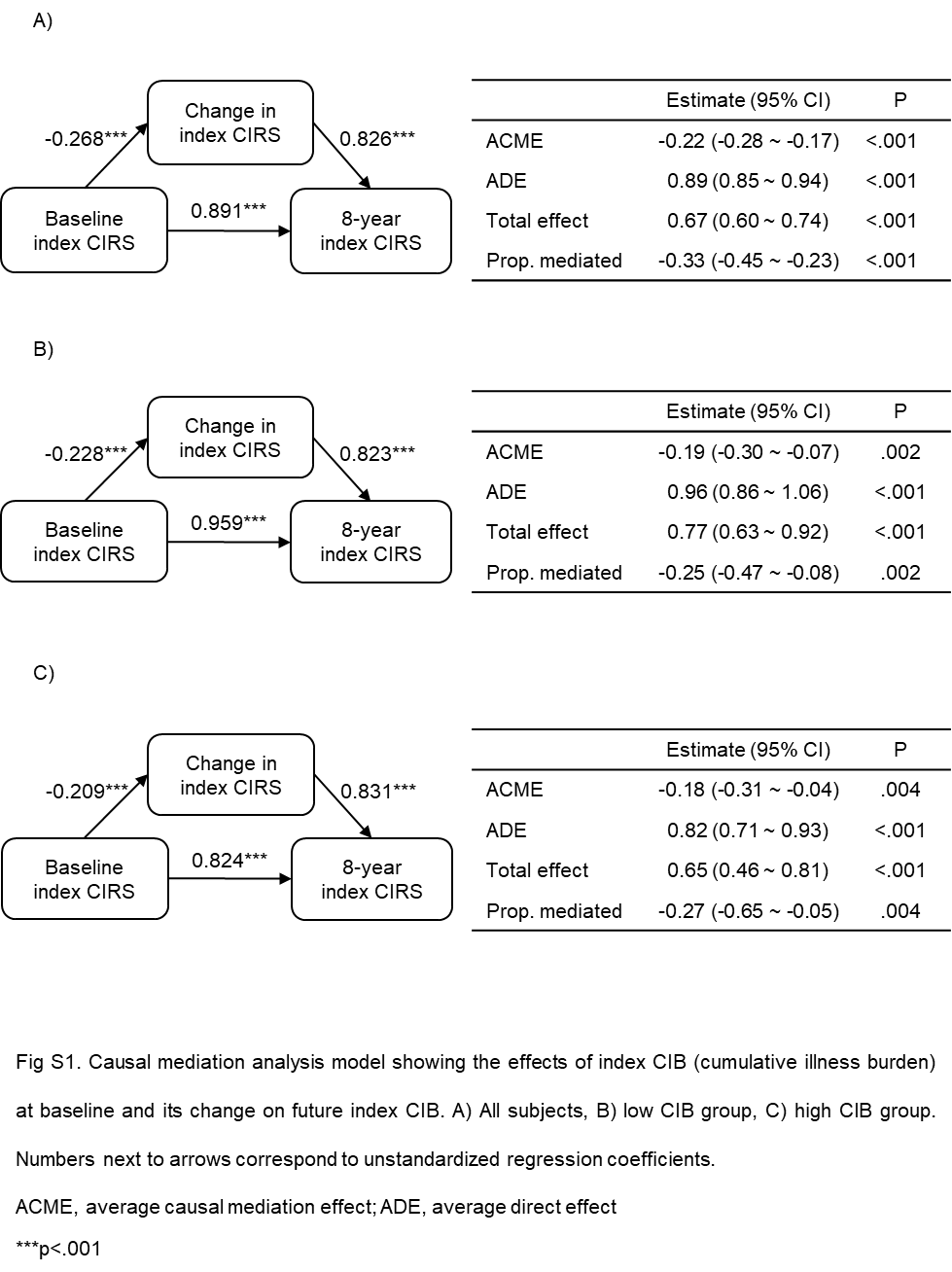

Supplement: Supplementary file 1 — Additional file 1: Table S1. Comparison of baseline demographic and clinical characteristics between the participants of the Korean Longitudinal Study on Cognitive Aging and Dementia who were invited to the current study as index cases and those who were not. Table S2. Comparison of demographic and clinical characteristics at the 8-year follow-up assessment between the couples included in the final analysis and those who were excluded. Figure S1. Causal mediation analysis model showing the effects of index CIB at baseline and its change on future index CIB. [file 12916_2023_3061_MOESM1_ESM.docx]
